# ClueDepth Grasp: Leveraging positional clues of depth for completing depth of transparent objects

**DOI:** 10.3389/fnbot.2022.1041702

**Published:** 2022-11-08

**Authors:** Yuanlin Hong, Junhong Chen, Yu Cheng, Yishi Han, Frank Van Reeth, Luc Claesen, Wenyin Liu

**Affiliations:** ^1^Guangdong University of Technology, Guangzhou, China; ^2^Hasselt University, Hasselt, Belgium

**Keywords:** depth completion, transparent objects, grasping, deep learning, robot

## Abstract

Obtaining accurate depth information is key to robot grasping tasks. However, for transparent objects, RGB-D cameras have difficulty perceiving them owing to the objects' refraction and reflection properties. This property makes it difficult for humanoid robots to perceive and grasp everyday transparent objects. To remedy this, existing studies usually remove transparent object areas using a model that learns patterns from the remaining opaque areas so that depth estimations can be completed. Notably, this frequently leads to deviations from the ground truth. In this study, we propose a new depth completion method [i.e., ClueDepth Grasp (CDGrasp)] that works more effectively with transparent objects in RGB-D images. Specifically, we propose a ClueDepth module, which leverages the geometry method to filter-out refractive and reflective points while preserving the correct depths, consequently providing crucial positional clues for object location. To acquire sufficient features to complete the depth map, we design a DenseFormer network that integrates DenseNet to extract local features and swin-transformer blocks to obtain the required global information. Furthermore, to fully utilize the information obtained from multi-modal visual maps, we devise a Multi-Modal U-Net Module to capture multiscale features. Extensive experiments conducted on the ClearGrasp dataset show that our method achieves state-of-the-art performance in terms of accuracy and generalization of depth completion for transparent objects, and the successful employment of a humanoid robot grasping capability verifies the efficacy of our proposed method.

## Introduction

Depth completion for transparent objects is a challenging problem in the field of computer vision because such objects have unique visual properties (e.g., reflection and refraction) that make them difficult to perceive by RGB-D cameras. To tackle this problem, classical methods (Klank et al., 2011; Alt et al., 2013) utilize RGB images from multiple views to infer position depth, however, these methods require long inference times and too many computational resources. To speed up the process, prior studies manually modified parameters (Ferstl et al., 2013; Ji et al., 2017; Guo-Hua et al., 2019) or exploited interpolation algorithms (Harrison and Newman, 2010; Silberman et al., 2012) to fill the holes in raw-depth images. However, it is difficult to restore objects' complex shape using these processes. Recently, with the development of deep learning, depth completion has received considerable attention from researchers in related fields.

The challenges of transparent object depth completion can be divided into two types, involves drifting point clouds caused by refraction, and the other involves missing point clouds caused by reflection. Hence, depth completion tasks also require correcting drifting points and adding missing points. Zhu et al. (2021) proposed a Local Implicit Depth Function for this purposed built using ray-voxel pairs that completed the missing depth using camera rays and their intersecting voxels. However, their method does not perform well for novel objects. To improve generalizability, Fang et al. (2022) devised Depth Filler Net that adapted dense blocks and a U-Net architecture to complete the missing depth. However, these methods suffer from missing local details and unclear outlines in the predicted depth images.

To acquire more 3D space features to complete the transparent area, Sajjan et al. (2020) extracted occlusion boundaries and surface normals from RGB images. Although these additional visual feature maps solved the problems of insufficient local details and blurred outlines, the global linear optimization function still requires too much computation. To this end, Tang et al. (2021) and Huang et al. (2019) proposed an encoder-decoder structure with an attention mechanism to improve training efficiency. Although these methods integrate different visual maps for completion, two problems persist. On the one hand, existing methods do not well-handle the reflection and refraction areas. Although they rely on deep learning methods to directly complete the depths or remove all transparent objects areas and reconstruct objects, deviations in the predicted depths commonly result. Notably, current state-of-the-art methods use a unified convolution algorithm to process different visual features, but they cannot obtain refined feature information.

In this paper, we propose the ClueDepth Grasp (CDGrasp)—deep learning approach for the depth completion of transparent objects. Compared with existing depth completion methods, ours analyzes the point clouds in the areas of transparent objects to remove drifted points while retaining correct points as clues for subsequent depth completion. Specifically, we first propose the ClueDepth module which uses the geometry method to remove drifted points that refract into the background, and we calculate the surface points of the missing features based on the object's contours. Then we filter the reflected points according to the reflection angle between the surface normal and the camera. The ClueDepth module thus directly provides the geometric details and position information for completion. We also design a DenseFormer network that integrates DenseNet (Iandola et al., 2014) and swin-transformer (Liu et al., 2021) blocks that expand the receptive fields and capture local fine-grained features and global information from RGB images. Because different modal visual maps contain distinct information, we propose a multi-modal U-Net module to distinguish the different visual features. The independent modal of module guarantees to obtain the acquisition of multiscale features without mutual interference from others, and the skip connection ensures that the multiscale features are fully leveraged in the decoder process, thus facilitating the generation of fine-grained depth maps.

In summary, our main contributions are as follows:

- We design an end-to-end CDGrasp deep-learning module that leverages the geometry method to filter-out the refractive and reflective points while preserving the correct points as positional clues for depth completion.
- We propose a DenseFormer network that combines DenseNet and swin-transformer blocks to extract local features and global information.
- We devise a multi-modal U-Net module that captures multiscale features from different visual maps and fuses them through skip connection to generate a fine-grained depth map.

Extensive experiments on the ClearGrasp dataset demonstrate that the proposed method outperforms state-of-the-art methods in terms of accuracy and generalization of depth completion for transparent objects. The successful grasping of transparent objects by a humanoid robot verifies the efficacy of our method, which will improve the robot's ability to perceive transparent objects in an actual production environment.

The remainder of this paper is organized as follows. Related works are reviewed in section Related work. The proposed method is described in detail in section Methodology. The experiments are described in Section Experiments. Finally, conclusions are presented in Section Conclusion and future work.

## Related work

### Depth completion

Depth completion aims to fill-in missing depth information by leveraging an existing depth map. Traditional works (Harrison and Newman, 2010; Silberman et al., 2012) primarily employ interpolation algorithms to do this, but they only consider the regular patterns of objects and have difficulty completing complex structures. Recently, deep-learning methods have demonstrated enormous potential for depth completion. For example, Xian et al. (2020) introduced an adaptive convolution method with three cascaded modules to address low-resolution and missing regions from indoor scenes. Hu et al. (2021) proposed a dual-branch convolutional neural network (CNN) that fuses a color image and sparse depth map to generate dense outdoor depths. Zhang and Funkhouser (2018) extracted surface normals and occlusion boundaries from RGB images as additional feature maps and utilized sparse Cholesky factorization to optimize the objective function to complete shiny, bright, and distant objects. Tang et al. (2021) devised a spectral residual block to deal with feature maps and took the lead in introducing a self-attentive adversarial network for depth completion, which achieved state-of-the-art performance with transparent objects. Although these methods exploit cross-modal visual maps as additional information, they simply concatenate the visual maps to extract features, leading to significant loss.

### Detecting transparent objects

Classical methods for detecting transparent objects mainly involve physical detection techniques (McHenry and Ponce, 2006; Maeno et al., 2013) that require specific equipment (Mathai et al., 2019) and lighting conditions (Fritz et al., 2009; Chu et al., 2018), resulting in difficult deployments in various environments. In contrast to classical methods, deep-learning methods can learn from large volumes of data to recognize transparent objects in different scenes. Zhang et al. (2021) designed a dual-head transformer for a transparency segmentation model that achieved joint learning from different datasets, successfully deploying it as a wearable system. Xu et al. (2021) proposed a real-time transparent object segmentation model that optimizes the atrous spatial pyramid pooling module by densely connecting atrous convolution blocks. Sajjan et al. (2020) estimated the 3D geometry of transparent objects by extracting multiple visual maps from a single RGB-D image and by driving a one-arm robot to pick up objects. However, they used transparent object masks to remove all transparent areas, which ignored the correct point cloud within those areas, resulting in the inaccuracy of predicted depths.

### Feature extraction

In the context of depth completion, prior works (Cheng et al., 2020; Park et al., 2020) used CNNs to extract coarse depth features and refined the structural details with spatial propagation networks. To overcome the limitations of the static CNN kernel, the authors in Huang et al. (2019), Tang et al. (2020), and Zhao et al. (2021) adopted content-adaptive CNNs for depth completion, which enhances network flexibility and accelerates computation. Recently, transformers have achieved outstanding performance on various computer vision tasks, such as object detection (Carion et al., 2020; Liu et al., 2021) and semantic segmentation (Strudel et al., 2021; Zheng et al., 2021). Ranftl et al. (2021) leveraged dense vision transformers to encode images from various vision transformer stages into tokens and reassembled them into image-like representations at various resolutions, thereby obtaining a global receptive field at each stage. Because the adoption of the transformer easily ignores local details, Yang et al. (2021) proposed TransDepth, which combines attention mechanisms and transformers to capture local details and long-range dependencies.

## Methodology

**Figure 1:**
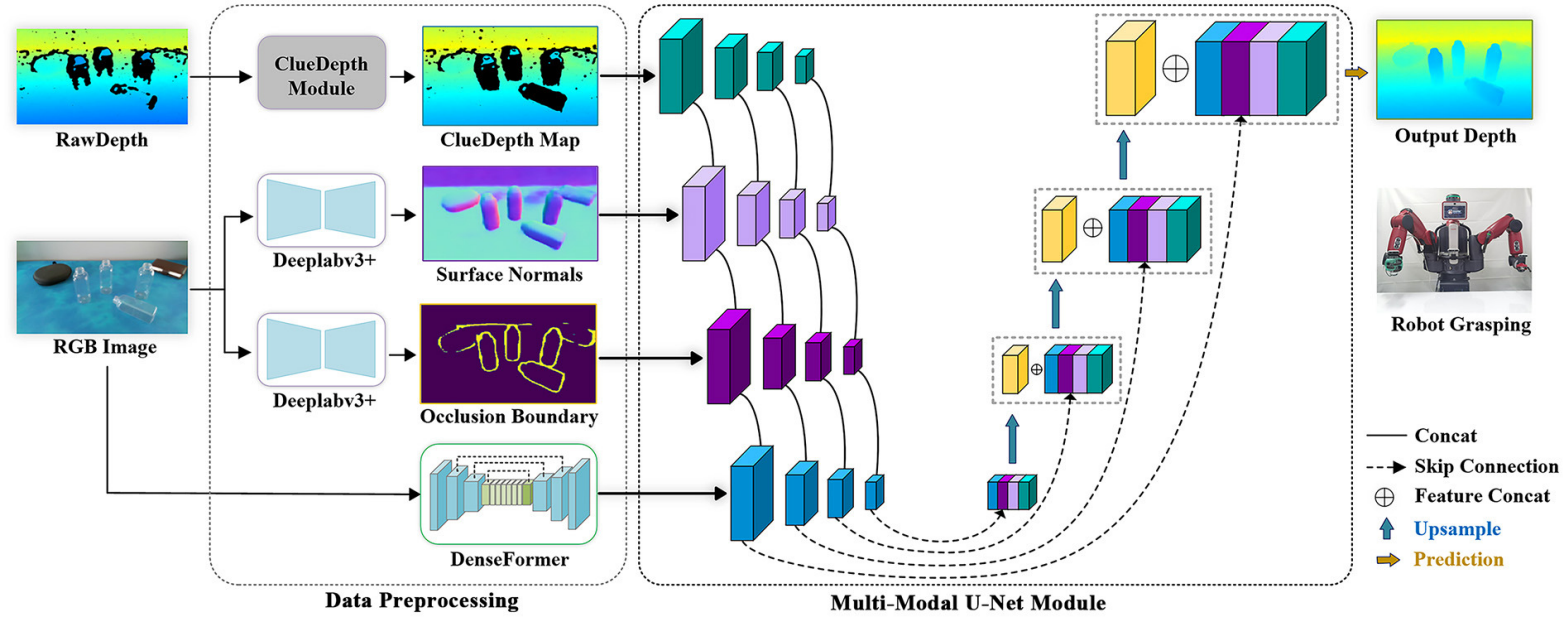
Overview of ClueDepth Grasp.

The proposed CDGrasp method is shown in Figure 1. Given an RGB-D image of transparent objects, we first extract the surface normals and occlusion boundaries from the RGB images, and our proposed ClueDepth module preserves the correct point clouds from the raw depths. In particular, for RGB images, we devised DenseFormer to extract both local and global features. These multi-modal visual maps are then input into our multi-modal U-Net module to extract multiscale features. Finally, the decoder with a skip connection fuses multiscale features and outputs the predicted depths.

### Data preprocessing

Surface normals and occlusion boundaries were verified by Sajjan et al. (2020) and Tang et al. (2021) as useful visual features for providing geometric object information. Surface normals can be used to reveal variations in lighting conditions, and occlusion boundaries can better distinguish object edges, thereby promoting the prediction of depth discontinuity boundaries. Therefore, we follow the same experimental setting as the work of Sajjan et al. (2020), which adopted Deeplabv3+ (Xu et al., 2021) with a DRN-D-54 backbone (Yu et al., 2017) to extract surface normals and occlusion boundaries from RGB images.

### ClueDepth module

**Figure 2:**
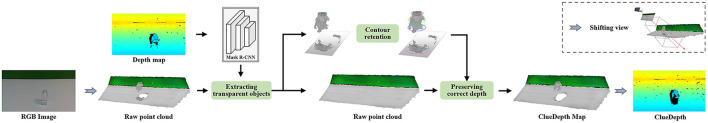
The overview of ClueDepth module.

To preserve the correct point cloud from raw depths ClueDepth module is employed, the overview of which is shown in Figure 2, where we first recognize the transparent objects and filter-out the background points. Subsequently, we retain the contours of objects to preserve their surface points. Finally, we preserve the correct points within a certain range of reflection angles. The details of the ClueDepth module are further presented in the following sub-subsections.

#### Extracting transparent objects

To locate the transparent objects, we first adopt a mask region-based (R)-CNN to recognize them from RGB images and map their correspondences to the depth maps. To obtain the depth values of transparent objects, we sample a number of points around the transparent objects and fit them into the planar equation, *z* = *C*_0_*x*+*C*_1_*y*+*C*_2_, where *z* represents the plane of the background, and *C*_0_, *C*_1_, and *C*_2_ represent the parameters of the plane expression. According to the equation, we calculate the distance, *h*_*m*_, from point *M* in the transparent object mask to the background plane:


(1) $$\begin{array}{l} h_{m}=\frac{\left(C_{0}x_{m}+C_{1}y_{m}+C_{2}{-z}_{m}\right)}{\sqrt{\left(C_{0}^{2}+C_{1}^{2}+{\left(-1\right)}^{2}\right)}},\;\left(x_{m},y_{m},z_{m}\right)\in M \end{array}$$

where *h*_*m*_ = 0 represents the points that are refracted into the background; thus, we filter these refracted points and keep points above the background, thus satisfying *h*_*m*_ > 0.

#### Contour retention

In addition to the points refracted into the background, we also must filter those refracted between the object surface and the background. Thus, we calculate the distance from points *T* to lens point *O*:


(2) $$\begin{array}{l} d_{t}=\sqrt{\left(x_{t}^{2}+y_{t}^{2}+z_{t}^{2}\right)-{\left(h_{0}{-h}_{t}\right)}^{2}},\;\left(x_{t},y_{t},z_{t}\right)\in T \end{array}$$

Because the light beams projected onto the surfaces of objects are distributed in an arc shape, we retain points *S*, whose distances fit the arc plane, and filter-out the refractive points between the surface of the object and the background.

#### Preserving the correct depth

Different camera angles have different reflection results, which have different effects on the preserved points. Taking the camera lens as the origin of the space coordinate axis, we define the incident ray, *l*_*p*_, which represents the vector from the object point cloud, *P*, to the lens point cloud, *O*, and denotes the object surface normal vector as *n*_*p*_. The surface normal vector is calculated using the depth variation of the point cloud with respect to its neighbors. The reflection angle, α_*p*_, between the incident ray, *l*_*p*_, and the surface normal vector, *n*_*p*_, is defined as follows:


(3) $$\alpha_{p}=\;arccos\frac{l_{p}\cdot n_{p}}{\left|l_{p}|\cdot |n_{p}\right|}$$

Typically, α_*p*_ is zero when light beams are projected perpendicular to the glass plane. As the reflection angle increases, two situations arise. First, the camera may become overexposed or underexposed when it does not receive the reflected light from the surface of the object, leading to missing depths. Second, the camera may receive light refracted to the background, resulting in an inaccurate depth value. Furthermore, at the thick edges of objects, light beams are projected to locations between the object's surface and the background, leading to inaccurate depth values. Accordingly, when the reflection angle, α_*p*_, is less than a certain angle, *K*, the camera can capture the correct point clouds from the depth map. Thus, we must verify a set of *K* angles to determine the best reflection conditions. Finally, we preserve the points that filter out refractions and reflections as clues for depth completion.

### DenseFormer

**Figure 3:**
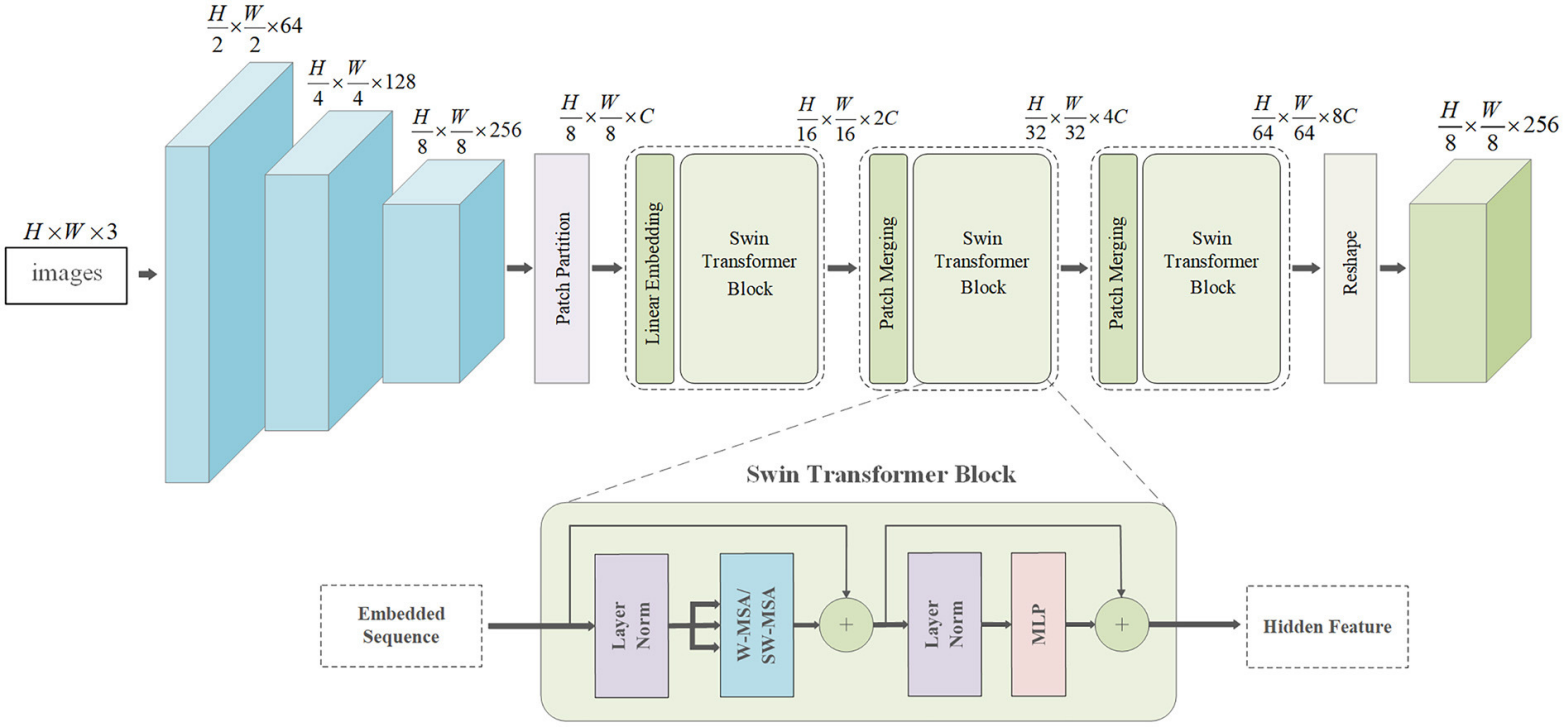
Structure of DenseFormer.

As a primary visual map, RGB images also contain intuitive non-visual information, such as the overall structure and local patterns of objects, which provide global and local features for transparent object completion. To this end, we propose the DenseFormer network to extract fine-grained features from RGB images. The network integrates DenseNet to extract local patterns underlying the image and the swin-transformer to enlarge the receptive field and acquire sufficient global information. Figure 3 illustrates the structure of the DenseFormer.

Given a three-channel RGB image, *I*, we first use DenseNet-121 pretrained on ImageNet (Krizhevsky et al., 2017) to extract features *x*,


(4) $$\begin{array}{l} x=DN\left(I\right),\;x\in {\mathbb{R}}^{H\times W\times 3} \end{array}$$

where *DN* denotes DenseNet-121, and *H* and *W* are the height and width of the images, respectively. The network consists of five blocks, each consisting of two adaptive convolutional layers and a leaky rectified linear unit layer. Based on the dense connections between layers, we can obtain fine-grained local features. However, limited by the receptive field of CNNs, DenseNet-121 cannot acquire sufficient global information to complete the depth map, especially for the overall structure of transparent objects. The transformer was verified to be effective in textual translation because of its attention mechanism, which captures large receptive fields. This motivated us to combine the transformer and DenseNet-121.

Specifically, tokenization is implemented by compressing the feature map, *x*, into a series of flat 2D patches $\left\{x_{t}^{i}\in {\mathbb{R}}^{T\times T\times C}|i=1,2,\ldots ,N\right\}$, where *T* × *T* represents the size of the patch, and $N=\frac{H\times W}{T^{2}}$ is the number of image patches. Subsequently, the 2D patches, *x*_*t*_, are mapped to the underlying D-dimensional embedding space via linear projection. To further encode the patch spatial information, we add specific positional embedding to preserve the positional information. The encoded image representation, *r*, is thus expressed as follows:


(5) $$\begin{array}{l} r=\left[x_{t}^{1}E;x_{t}^{2}E;\ldots ;x_{t}^{N}E\right]+E_{pos} \end{array}$$

where *E*∈ℝ^(*T*×*T*×*C*)×*D*^ is the patch embedding projection, and $E_{pos}\in {\mathbb{R}}^{N\times D}$ denotes the position embedding. Thus, the swin-transformer blocks can be formulated as


(6) $$\begin{array}{l} r_{\ell }^{\prime }=MSA(LN(r_{\ell -1}))+r_{\ell -1} \end{array}$$

(7) $$\begin{array}{l} r_{\ell }=MLP(LN(r_{\ell }^{\prime }))+r_{\ell }^{\prime } \end{array}$$

where *MSA* denotes the multi-head self-attention of windows, *MLP* denotes the Multi-Layer Perceptron, *LN*(•) denotes the layer normalization operator, and *r*_ℓ_ represents the encoded image representation. Finally, the feature of the last transformer layer, *r*_*L*_, is reshaped to *x*′ ∈ ℝ^*H*×*W*×*C*^ for subsequent CNN decoding.

### Multi-modal U-net module

Effectively leveraging additional visual feature maps can suitably complement the necessary details for depth completion (e.g., transparent object geometry and lighting conditions). However, existing approaches only concatenate visual maps and adopt unified convolutions for encoding (Huang et al., 2019; Tang et al., 2021; Fang et al., 2022), which cannot make full use of visual information. Moreover, the missing regions and proportions of transparent objects critically affect the performance of convergence algorithms (e.g., batch normalization), which require mean and variance operators (Zhu et al., 2021).

To this end, we propose a multi-modal U-Net module to capture multiscale features from different modal visual maps. This module has four inputs (i.e., RGB image, ClueDepth map, surface normals, and occlusion boundaries), in which each input is encoded separately. For example, for the RGB image, we deploy the DenseFormer network to extract features, and for the rest of the visual maps, we construct five downsampling blocks to extract them, where each downsampling block consists of two convolutional layers and one average pooling layer.

For the decoder, we concatenate the encoded features and adopted a skip connection to complement the low-level features for network fusion. Specifically, we denote the encoded features of RGB images as $\phi_{R}^{L}$, the ClueDepth map as $\phi_{C}^{L}$, the surface normal as $\phi_{N}^{L}$, the occlusion boundary as $\phi_{B}^{L}$, and the fused features as ϕ^*L*^, where *L* denotes the layer. The decoder process is formulated as follows:


(8) $$\varphi^{L}=\{\begin{array}{c} g(f(\phi_{R}^{L-1},\phi_{C}^{L-1},\phi_{N}^{L-1},\phi_{B}^{L-1}\;)),\;L=1\\ g(f(\phi_{R}^{L-1},\phi_{C}^{L-1},\phi_{N}^{L-1},\phi_{B}^{L-1},\phi^{L-2}\;)),\;L>1 \end{array}$$

where *f* denotes feature concatenation, and *g* represents upsampling. Finally, the network outputs a complete depth map of transparent objects.

## Experiments

In this section, we introduce the details of the dataset and experimental settings and evaluate the performance of CDGrasp in both synthetic and real-world environments. Finally, we verify our system using a real robot grasping task.

### Dataset

**Figure 4:**
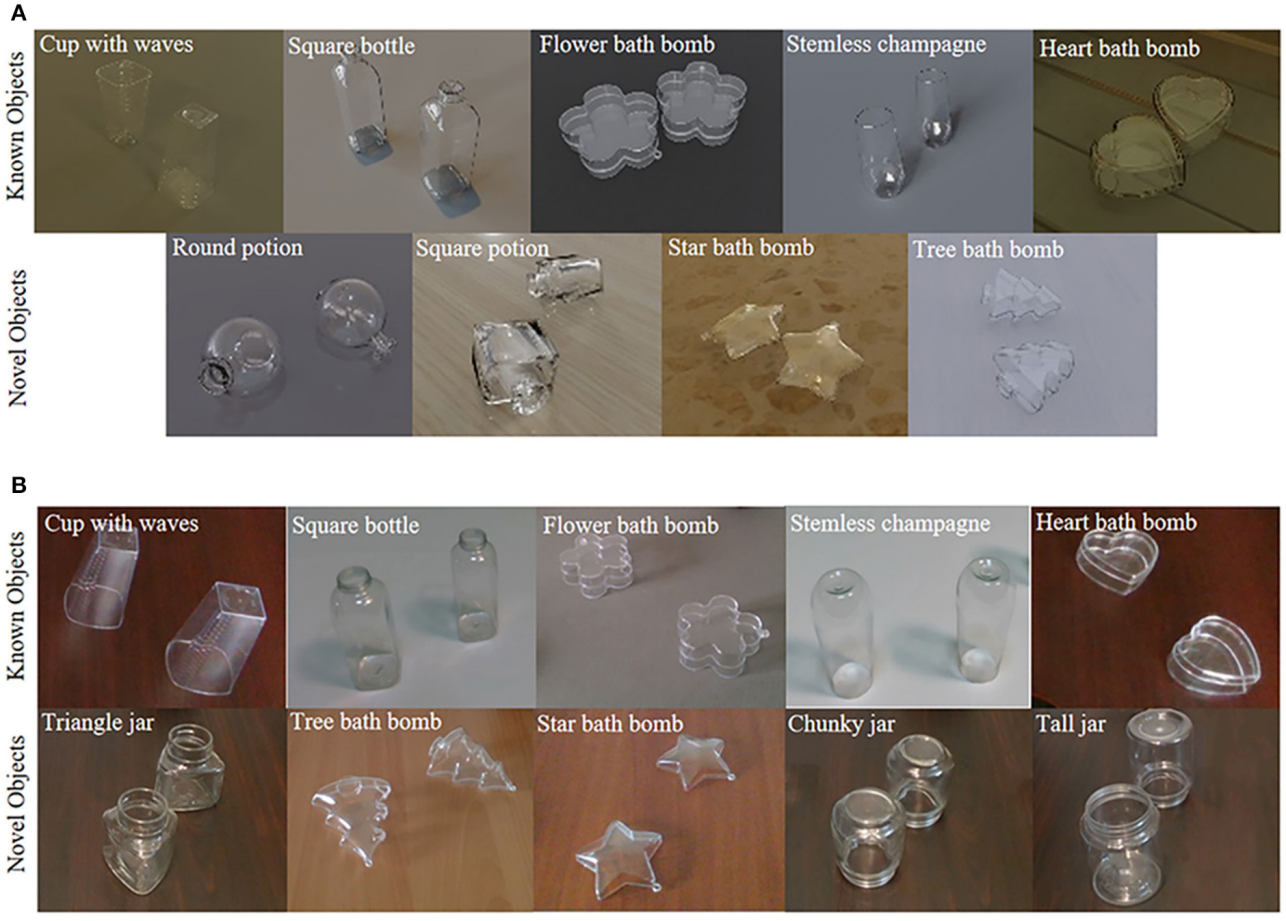
Examples of transparent objects in ClearGrasp dataset. **(A)** Known and novel objects in Synthetic dataset. **(B)** Known and novel objects in the real-world dataset.

The ClearGrasp dataset is a publicly available transparent object dataset that contains more than 50k transparent object images, including 9 classes of synthetic objects and 10 classes of real-world objects. As shown in Figure 4, the objects are further classified into known and novel types. The data divisions follow the settings in Sajjan et al. (2020), from which five known synthetic objects are used for training, and five overlapping real-world objects are used for testing. To verify the generalizability, four novel synthetic objects and five novel real-world objects were also used for testing.

### Evaluation metrics

Following Huang et al. (2019), Sajjan et al. (2020), and Tang et al. (2021), a number of metrics are adopted to evaluate the performance of depth completion, including the root mean square error (RMSE), mean absolute error (MAE), absolute relative difference (REL), and threshold accuracy. Threshold accuracy is denoted as δ_*t*_, and threshold *t* is set to 1.05, 1.10, and 1.25. Among them, δ_1.05_ has the strictest requirements on completion accuracy, which can better reflect the overall accuracy of completion.

### Baseline approaches

We compare our method to other baseline approaches, which consist of the following traditional algorithms and deep-learning methods:

- **Joint bilateral filter (JBF)**. A principled approach (Silberman et al., 2012) is applied to infer physical relationships and repair holes using an interpolation algorithm based on the JBF.
- **Anisotropic diffusion (AD)**. A second-order smoothness term (Harrison and Newman, 2010) is used to extrapolate both planar and curved surfaces.
- **Decoder modulation (DM)**. An additional decoder branch (Senushkin et al., 2021) that considers missing depth values is used as input, and the mask distribution is adjusted to improve accuracy.
- **ClearGrasp (CG)**. Surface normals, mask transparent surfaces, and occlusion boundaries are exploited (Sajjan et al., 2020) to infer the accurate depths of transparent objects.
- **DepthGrasp (DG)**. A self-attentive adversarial network (Tang et al., 2021) is used to capture the structural information of a transparent object and achieve the best results.

### Depth completion performance

Table 1 lists the performance results of different depth completion methods, from which we can see that, compared with traditional methods including JBF and AD, the deep learning methods obtain significant improvements in performance, because traditional methods only interpolate missing depths based on object edges, which ignores global information. The DM approach completes the basic shape of the object, which is very important for robot grasping and positioning. However, it cannot handle complex structures owing to the lack of local geometric details. Although CG and DG achieve competitive results with additional geometric information and global optimization functions, they lack the original correct raw depth information to provide positional clues, leading to deviations in predicted depth. Our proposed method achieves state-of-the-art results owing to the ClueDepth module, which preserves the correct location information, and the DenseFormer with the multi-modal U-Net module, which captures the geometric structure of transparent objects.

**Table 1:** Comparison of depth completion performance.

	**Error metrics**			**Accuracy metrics**		
**Model**	**RMSE↓**	**REL↓**	**MAE↓**	**δ_1.05_↑**	**δ_1.10_↑**	**δ_1.25_↑**
JBF (Fritz et al., 2009)	0.389	0.53	0.358	27.61	37.28	51.32
AD (Ferstl et al., 2013)	0.315	0.489	0.297	41.26	61.29	71.24
DM (Yu et al., 2017)	0.049	0.075	0.038	59.67	75.85	95.96
CG (He et al., 2016)	0.038	0.048	0.027	72.94	87.88	97.17
DG (Hu et al., 2021)	0.031	0.039	0.021	74.69	89.73	97.35
**Ours**	**0.022**	**0.026**	**0.019**	**80.16**	**94.82**	**98.64**

**Figure 5:**
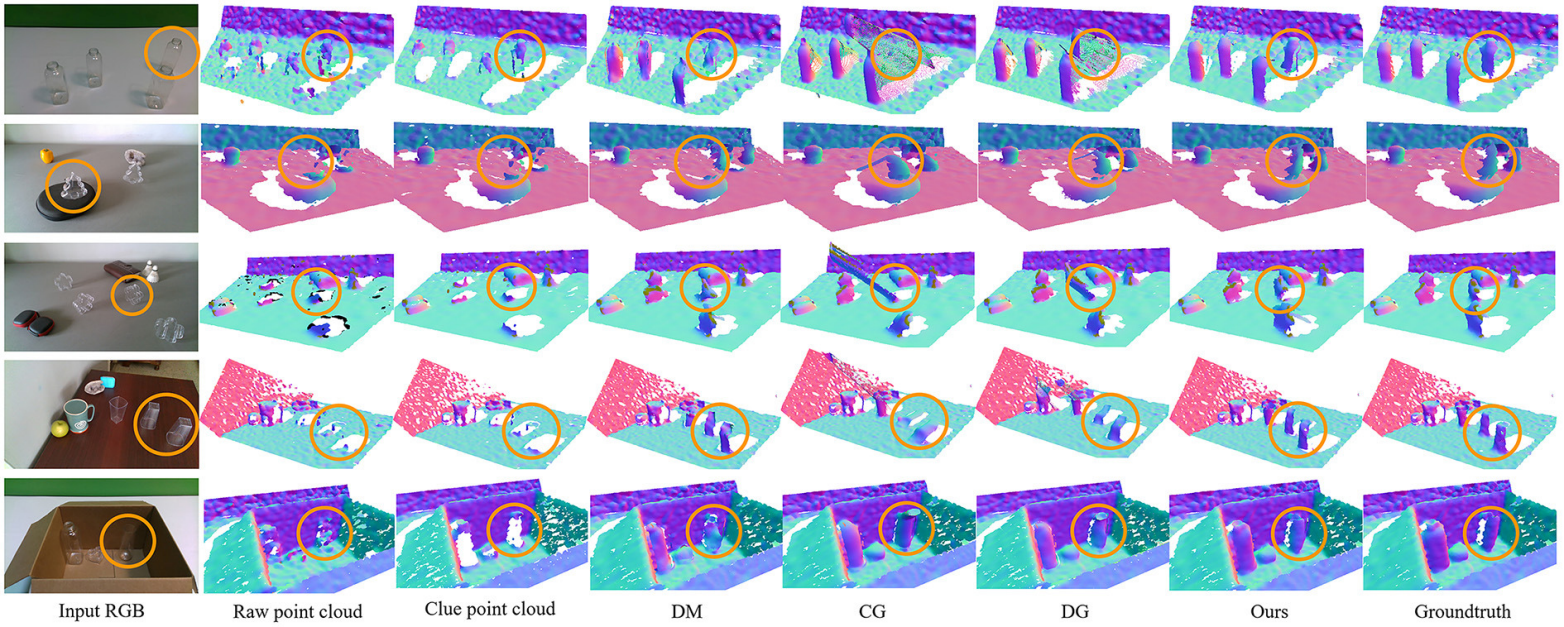
Qualitative comparison of the approaches.

We intuitively visualize some examples of the completed depth maps in Figure 5. Where “Raw point cloud” is the rawdepth obtained by switching the 3D view, and “ours” represents the fine-grained depth maps generated by CDGrasp. The orange circles indicate that CDGrasp filters-out the reflective and refractive areas and retains the correct raw depth information for locating objects. The contrast between the red circles indicates that our method can precisely complete the missing depth with a clear shape.

### Evaluation of reflection angle K

**Figure 6:**
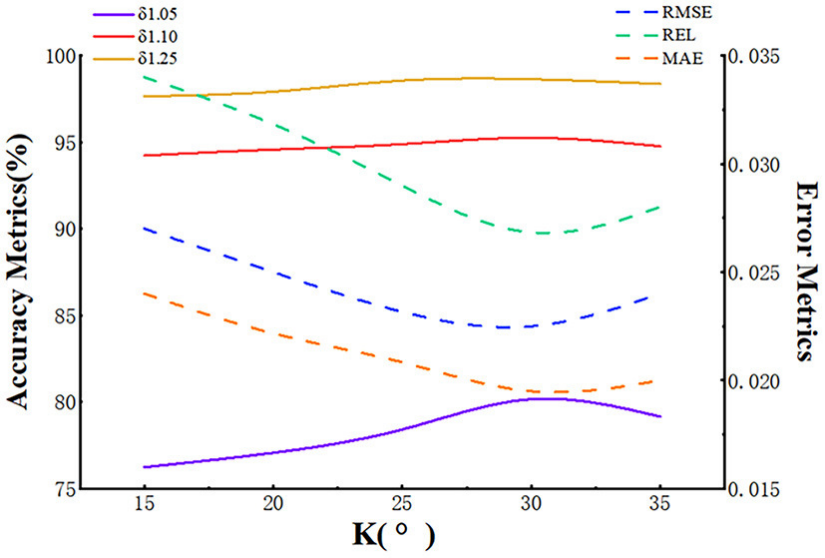
Evaluation of the reflection angle K.

The reflection angle determines the reflectivity of the object's surface, which critically affects the retention of clue points. Thus, we conducted quantitative experiments that included five sets of different angles at gradients of 5°. The results are shown in Figure 6, which shows the effect of different *K*-value settings on the completion performance. It can be seen that as the reflection angle increases, the accuracy has a certain degree of improvement as it benefits from the enlargement of local areas, which provide more in-depth information. However, when the reflection angle reaches 30°, the accuracy decreases slightly because the light beams are reflected by the surfaces of the object, leading to a loss of depth values.

### Ablation studies

To illustrate the effectiveness of each component, we conducted a series of ablation experiments, as described in the following sub-subsections.

#### Effect of DenseFormer

For comparison, we used ResNet18 (He et al., 2016) as the network backbone to demonstrate the module's effects. The results are listed in Table 2, from which we can see that DenseNet performs slightly better than ResNet18 owing to the dense connection between each layer and the reuse of features. By combining the transformer, the method achieves a significant improvement because the transformer expands the receptive field and obtains global information for completing depth maps.

**Table 2:** Effectiveness of DenseFormer of CDGrasp.

	**Error metrics**			**Accuracy metrics**		
**Model**	**RMSE↓**	**REL↓**	**MAE↓**	**δ_1.05_↑**	**δ_1.10_↑**	**δ_1.25_↑**
ResNet18	0.028	0.040	0.022	76.25	92.19	98.74
DenseNet	0.027	0.037	0.021	78.12	94.41	**98.93**
DenseFormer	**0.022**	**0.026**	**0.019**	**80.16**	**94.82**	98.65

#### Effect of the multi-modal U-net module

For clarity, “Concat” indicates that all maps are concatenated and sent into encoder for unified encoding, while “Multi-Modal” means that the visual maps are encoded through multi-modal U-Net module. As shown in Table 3, the multi-modal U-Net module significantly improves performance compared with the concatenation method, mainly because the multi-modal U-Net module guarantees the extraction of multiscale features from each modal maps and fuses the features through the skip connection.

**Table 3:** Effectiveness of multi-head encoder of CDGrasp.

	**Error metrics**			**Accuracy metrics**		
**Model**	**RMSE↓**	**REL↓**	**MAE↓**	**δ_1.05_↑**	**δ_1.10_↑**	**δ_1.25_↑**
Concat	0.024	0.038	0.021	77.43	**94.94**	98.63
Multi-modal	**0.022**	**0.026**	**0.019**	**80.16**	94.82	**98.64**

### CDGrasp generalization

Table 4 presents the generalizability of the proposed model to real-world images and novel objects. These images are from the cleargrasp dataset. From the table, we can see that the proposed model generalizes remarkably well for both synthetic and real-world objects. In particular, in terms of known synthetic objects, our method achieves an improvement of better than 5%, benefitting from the multi-modal U-Net module structure and the DenseFormer, which captures 3D geometric structures. In terms of synthetic novel objects, the performance drops slightly because the synthetic novel objects (e.g., star- and tree-shaped) are more irregular than the known objects. Our method also has a critical performance improvement on real-world objects because, in real-world environments, reflection and refraction phenomena will be more obvious, and our ClueDepth module prevents this from creating incorrect points while preserving the correct ones as positional guidance features for completing the depth map. From Figure 5, we can see that completion errors mainly occur in overlapping and distant regions because the surfaces of transparent objects are difficult to capture when they are too distant from the camera or obscured, which causes the network to inaccurately extract structural features.

**Table 4:** CDGrasp generalizes to both real-world images and novel transparent objects on depth completion.

**Methods**	**Error metrics**			**Accuracy metrics**		
**Model**	**RMSE↓**	**REL↓**	**MAE↓**	**δ_1.05_↑**	**δ_1.10_↑**	**δ_1.25_↑**
		**ClearGrasp synthetic-known**				
CG (He et al., 2016)	0.044	0.047	0.033	71.23	92.6	98.24
RVP (Guo-Hua et al., 2019)	0.034	0.045	0.026	73.53	92.68	98.25
DG (Hu et al., 2021)	0.037	0.037	0.030	75.19	92.97	98.79
Ours	**0.021**	**0.028**	**0.018**	**84.93**	**96.11**	**99.03**
		**ClearGrasp synthetic-novel**				
CG (He et al., 2016)	0.04	0.071	0.035	42.95	80.04	98.10
RVP (Guo-Hua et al., 2019)	0.037	0.062	0.032	50.27	84.00	**98.39**
DG (Hu et al., 2021)	0.039	**0.059**	0.034	51.86	82.14	98.23
Ours	**0.035**	0.065	**0.032**	**56.73**	**87.66**	98.32
		**ClearGrasp real-known**				
CG (He et al., 2016)	0.039	0.053	0.029	70.23	86.98	97.25
RVP (Guo-Hua et al., 2019)	0.032	0.042	0.024	74.63	90.69	98.33
DG (Hu et al., 2021)	0.031	0.039	0.021	74.69	89.73	97.35
Ours	**0.022**	**0.026**	**0.019**	**80.16**	**94.82**	**98.64**
		**ClearGrasp real-novel**				
CG (Sajjan et al., 2020)	0.028	0.04	0.022	79.18	92.46	98.19
RVP (Zhu et al., 2021)	0.027	0.039	0.022	79.50	93.00	99.28
DG (Tang et al., 2021)	0.022	0.033	**0.017**	82.37	93.46	98.48
Ours	**0.021**	**0.030**	0.018	**83.67**	**95.06**	**99.12**

### Robot grasping

**Figure 7:**
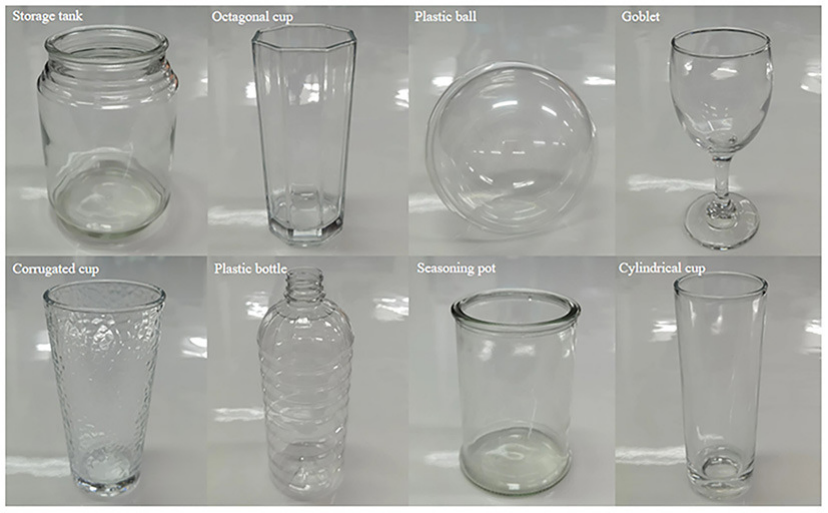
Real novel objects for grasping.

To verify the practical use of the proposed method, we deployed CDGrasp on a humanoid robot, Baxter, so that it would grasp real-world transparent objects. In particular, we used the GR-CNN (Kumra et al., 2020), which was verified by Tang et al. (2021) as a good grasp detection method. We chose eight novel transparent objects that do not overlap with ClearGrasp as our tested objects, including “Storage tank,” “Octagonal cups,” ”Plastic ball,” “Goblet,” “Corrugated cup,” “Plastic bottle,” “Seasoning pot,” and “Cylindrical cup.” These objects are shown in Figure 7.

**Figure 8:**
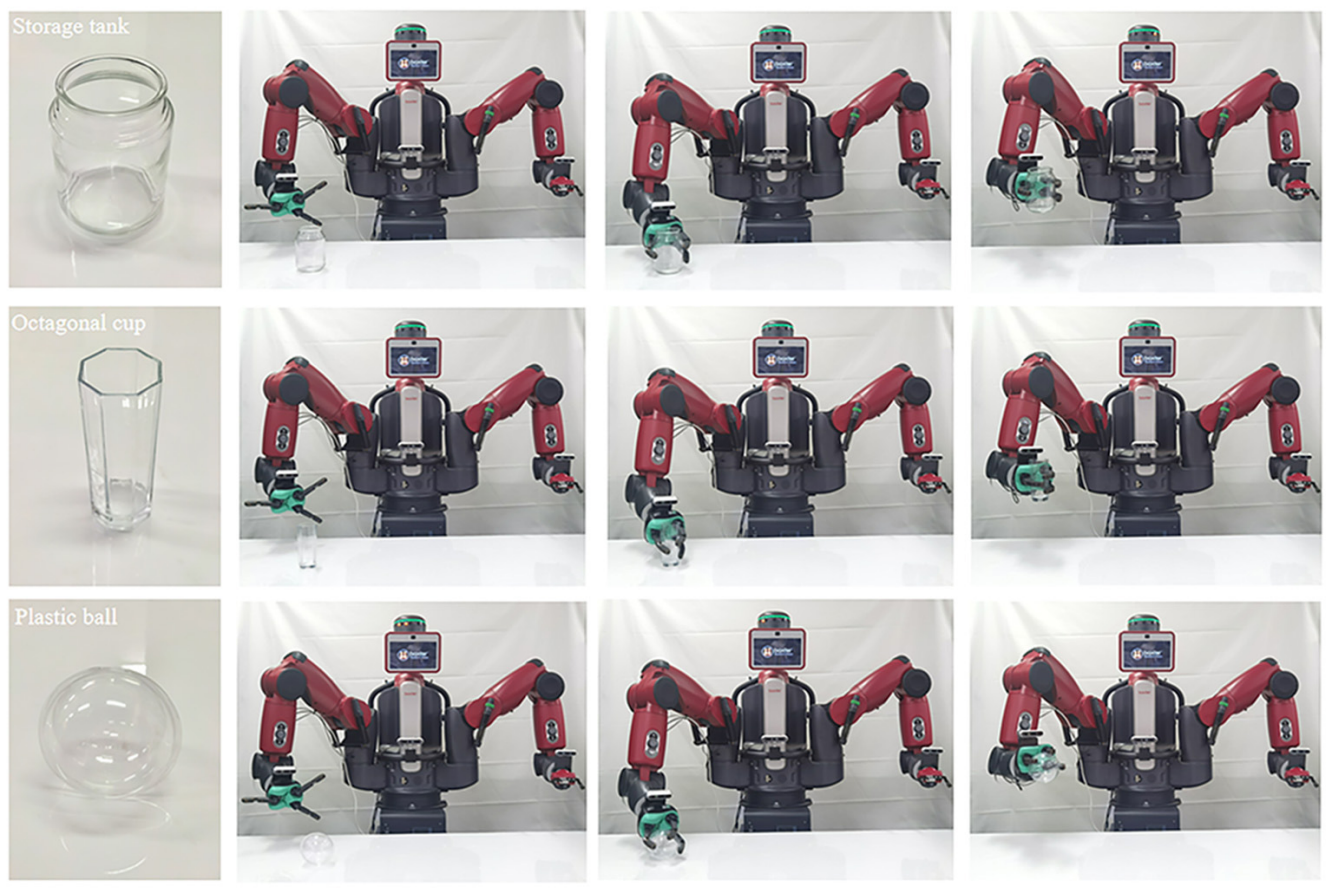
Examples of grasping novel transparent objects by Baxter using our proposed system.

Figure 8 presents examples of robots grasping transparent objects. The Baxter robot used in the experiments generates grasping strategies based on the deployed modules. The actual crawling environment uses only a white background table. For each object, Baxter tries to grasp it 10 times, and the success rate depends on whether it holds the object for more than 10 s. This setting avoids falling after a short-term grab from being judged as a successful grab. Table 5 summarizes the success rate, from which we can see that it does so with high accuracy based on our depth completion method. It outperforms the state-of-the-art method DepthGrasp (Tang et al., 2021) method, demonstrating the efficacy of our method.

**Table 5:** Novel object grasping in the real-world environment.

**Transparent objects**	**Rawdepth**	**DepthGrasp**	**CDGrasp**
Storage tank	2/10	7/10	8/10
Octagonal cup	5/10	8/10	9/10
Plastic ball	2/10	5/10	7/10
Goblet	1/10	8/10	9/10
Corrugated cup	4/10	9/10	10/10
Plastic cup	3/10	9/10	9/10
Seasoning pot	4/10	8/10	9/10
Cylindrical cup	5/10	8/10	9/10
Success rate (%)	**32.5**	**77.5**	**87.5**

## Conclusion and future work

In this study, we proposed a novel depth-completion model for transparent objects. Specifically, we proposed the ClueDepth module, which preserves the correct depth values and directly provides 3D geometric clues for positional guidance. We then devised a DenseFormer network that integrates DenseNet and swin-transformer blocks to extract local features and expand the receptive fields for global information acquisition. To fully exploit different visual maps, we proposed a multi-modal U-Net module to extract multiscale features from visual maps separately. Extensive experiments demonstrated that our method achieved state-of-the-art results in terms of accuracy and generalizability. Among them, for the depth completion of transparent objects in real scenes, our method improves the δ_1.05_ performance by 5.47%.

Based on the correct point cloud on the transparent object, the adaptive method of retaining the correct point is a worthy future research direction, and the experiment should be extended to more complex objects as much as possible.

## Data availability statement

Publicly available datasets were analyzed in this study. This data can be found here: https://sites.google.com/view/cleargrasp/data?authuser=0.

## Author contributions

YHo and JC are responsible for the experiments and writing. YC, YHa, FR, LC, and WL are responsible for the experimental and writing guidance.

## Funding

This work is supported by the National Natural Science Foundation of China (No. 91748107, No. 62076073, No. 61902077), the Guangdong Basic and Applied Basic Research Foundation (No. 2020A1515010616), Science and Technology Program of Guangzhou (No. 202102020524), the Guangdong Innovative Research Team Program (No. 2014ZT05G157), Special Funds for the Cultivation of Guangdong College Students' Scientific and Technological Innovation (pdjh2020a0173), and the Key-Area Research and Development Program of Guangdong Province (2019B010136001), and the Science and Technology Planning Project of Guangdong Province LZC0023.

## Conflict of interest

The authors declare that the research was conducted in the absence of any commercial or financial relationships that could be construed as a potential conflict of interest.

## Publisher's note

All claims expressed in this article are solely those of the authors and do not necessarily represent those of their affiliated organizations, or those of the publisher, the editors and the reviewers. Any product that may be evaluated in this article, or claim that may be made by its manufacturer, is not guaranteed or endorsed by the publisher.
